# Hepatic emphysema associated with ultrasound-guided liver biopsy in a dog

**DOI:** 10.1186/1751-0147-56-25

**Published:** 2014-04-23

**Authors:** Frida Westgren, Tove Hjorth, Margareta Uhlhorn, Pernille E Etterlin, Charles J Ley

**Affiliations:** 1 Department of Diagnostic Imaging, University Animal Hospital, Swedish University of Agricultural Sciences, Box 7040, Uppsala 75007, Sweden; 2 Division of Pathology, Pharmacology and Toxicology, Department of Biomedical Sciences and Veterinary Public Health, Swedish University of Agricultural Sciences, Uppsala 75007, Sweden; 3 Department of Clinical Sciences, Swedish University of Agricultural Sciences, Uppsala 75007, Sweden

**Keywords:** Septicemia, Bacteriemia, Complication, *Clostridium perfringens*, Gas

## Abstract

An eleven-year-old Chinese Crested Powder Puff dog presented with polydipsia/polyuria, inappetence, diarrhea and vomiting underwent an ultrasound-guided percutaneous liver biopsy. Two days post-biopsy the clinical condition of the dog acutely deteriorated with fever, dyspnea, ataxia and subcutaneous emphysema. Radiographs and ultrasound showed focal severe hepatic emphysema in the region of the previous liver biopsy. Post-mortem examination revealed chronic hepatitis with dissecting fibrosis, acute hepatitis with hemorrhage and in the hindlimb musculature extensive hemorrhage and necrosis. Pure cultures of the gas producing bacteria *Clostridium perfringens* were isolated in samples from the hind limb musculature. We propose that the hepatic emphysema in the region of the biopsy site was a result of a clostridial infection.

## Background

Liver biopsy, including both ultrasound-guided and blinded techniques, is considered a safe procedure with reported major complication rates between 0.01-5.9% of the cases in humans [1,2] and low complication rates in dogs [3,4]. In humans hemorrhage is the most commonly encountered major complication (0.2-1.7% of patients) and biliary peritonitis, hemobilia, hypotension, arteriovenousfistula, pulmonary embolism and ileus occur in less than 1% of the cases [1,2]. Transient bacteriemia associated with liver biopsy has been shown with blood and liver tissue culture to have an incidence of 2-13%, but clinically evident infectious complications are considered extremely rare [1,5]. The majority of the patients with liver biopsy associated bacteriemia have pathological changes consistent with hepatocellular or hepatobiliary disease [1,5]. Furthermore defects in the patients defence mechanisms occur due to liver disease including deficiencies of serum complement, inhibition of chemotaxis, colonization of the small bowel by enteric organisms and bypass of bacterial clearance mechanisms via intrahepatic shunts [6,7]. We present a case of hepatic emphysema associated with ultrasound-guided liver biopsy and possible clostridial bacteriemia.

## Case presentation

### Signalment, history and clinical findings

An 11-year-old male Chinese Crested Powder Puff was presented with progressive polyuria, polydipsia and inappetence. The general physical examination was normal except for a moderate systolic heart murmur. Biochemical tests revealed elevations of alanine aminotransferase (282 IU/l) [ref 0-78 IU/l], alkaline phosphatase (1812 IU/l) [ref 0-132 IU/l] and bile acids (14.7 μmol/l) [ref 0-12 μmol/l].

### Imaging, diagnosis and outcome

**Figure 1:**
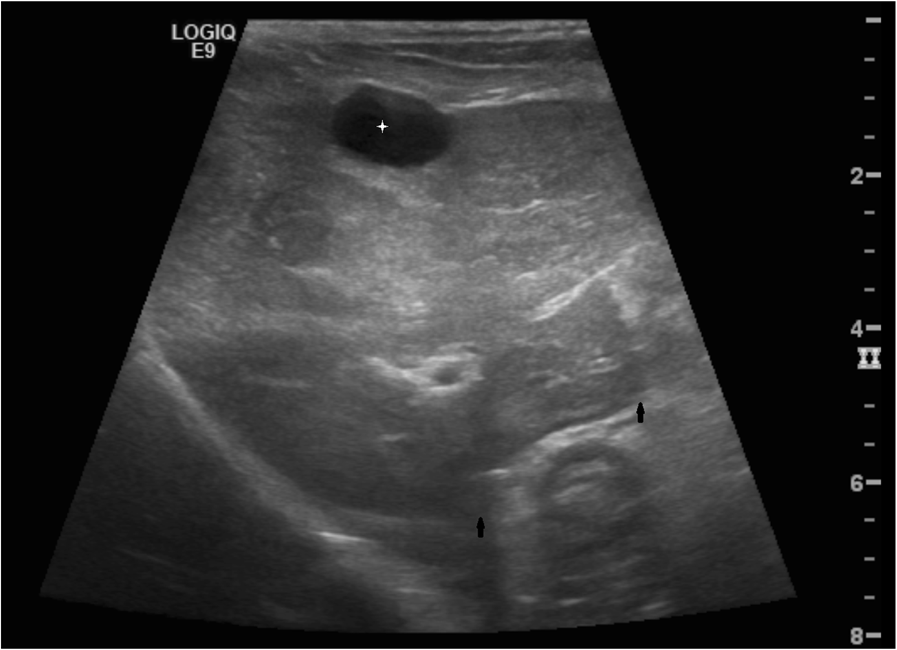
**Initial hepatic ultrasonographic image.** An oblique plane is used with a ventral acoustic window, showing part of the gallbladder (white star), rounded liver margin (black arrow) and heterogenous parenchyma.

Abdominal ultrasonography, using a high-frequency transducer (9 MHz linear probe, GE Medical LOGIQ 9, General Electric Medical Systems, Wisconsin, USA), was done with the dog in dorsal recumbency and unsedated. The liver was considered mildly enlarged with rounded margins and in all liver lobes focal rounded variably sized areas of mixed echogenicity and smaller hypoechoic areas were seen (Figure 1). Differential diagnosis considered were hepatitis, steroid hepatopathy, lymphoma, hepatocellular carcinoma, metastasis and amyloidosis, possibly associated with nodular hyperplasia. A mild homogenous echoic pattern was seen in the dependant portion of the gallbladder with some smaller (approximately 5 mm) more echoic non-acoustic shadowing structures in the region of the neck of the gallbladder. This was considered most likely to be a suspension of variably concentrated bile pigment precipitates (sludge) and cholestasis was considered as a possible cause, but there was no sign of obstruction of the bile ducts. Both kidneys had normal size and shape but had increased echogencity of the cortex and medulla and a mildly decreased corticomedullary definition. Several small rounded hypoechoic structures most likely representing nodular hyperplasia or neoplasia were seen in the right pancreatic lobe.

Ultrasound-guided percutaneous liver biopsy was scheduled for the following week. Diarrhea and one episode of vomiting were observed on the day of the planned biopsy procedure but immediately prior to the biopsy procedure the clinical condition of the dog was considered stable and the dog had normal temperature, thus the biopsy was performed as planned.

A standard anesthetic protocol including propofol induction and isoflurane maintenance was used. Two free-hand ultrasound-guided percutaneous liver biopsies (18 gauge needle, BARD biopsy needle and BARD magnum biopsy instrument, Crawley, UK) using a longitudinal plane and targeting two of the focal heterogenous areas of the liver via a left cranial abdominal window were taken. The biopsy needle position was maintained to the left and dorsal to the gallbladder and the gallbladder avoided during penetration of the liver and firing of the biopsy device. No complications were detected during the procedure or when the biopsy region was scanned immediately after the procedure. The dog was discharged the following day since it was reported to be bright and alert with normal temperature and only one occasion of diarrhea and vomit had occurred post-biopsy.

The dog was returned to the clinic two days later when the owner reported that the diarrhea and vomiting had increased in frequency and the general condition of the dog had acutely deteriorated. Clinical examination revealed fever, dyspnea, ataxia and subcutaneous emphysema. Laboratory tests showed neutrophilia with moderate left-shift, hundredfold increased value of C-reactive protein and a mixed metabolic acidosis and respiratory alkalosis (HCO_3_^−^ 15.6 mmol/l [ref 22.2-27.2 mmol/l] and PCO_2_ 19.1 kPa [31.8-54 kPa]).

**Figure 2:**
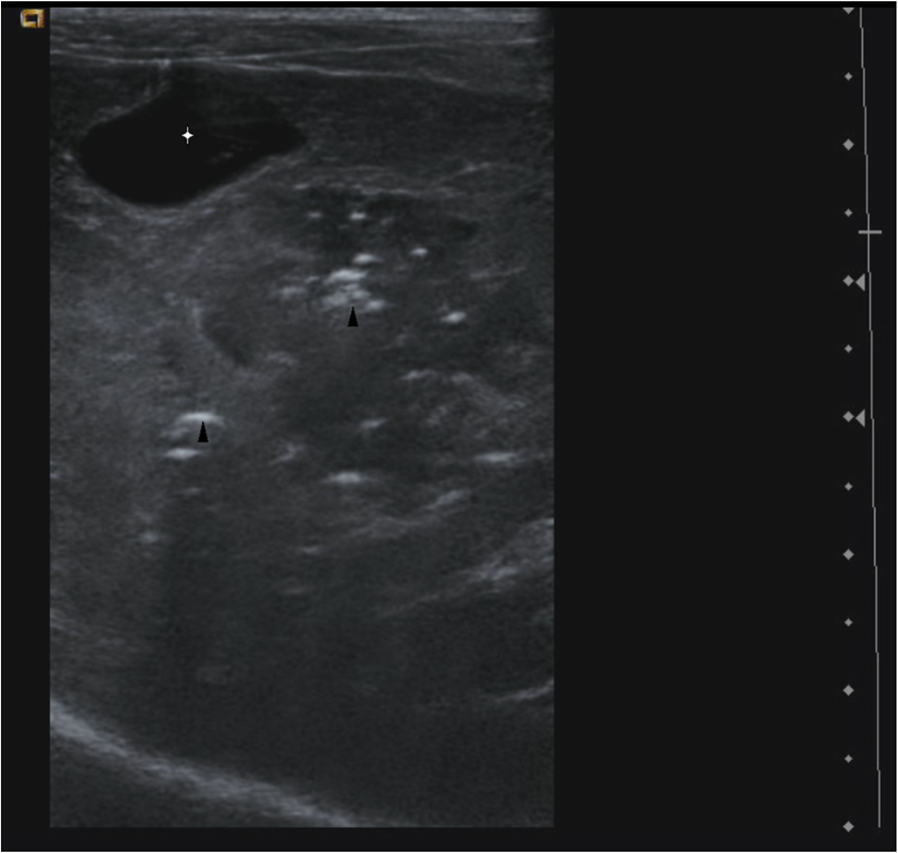
**Two – days post-biopsy hepatic ultarsonographic image.** Approximately the same plane is used and the same acoustic window as Figure 1, revealing echoic gas foci (black arrowheads) at the previous biopsy site. Gallbladder (white star) shows a normal appearance.

**Figure 3:**
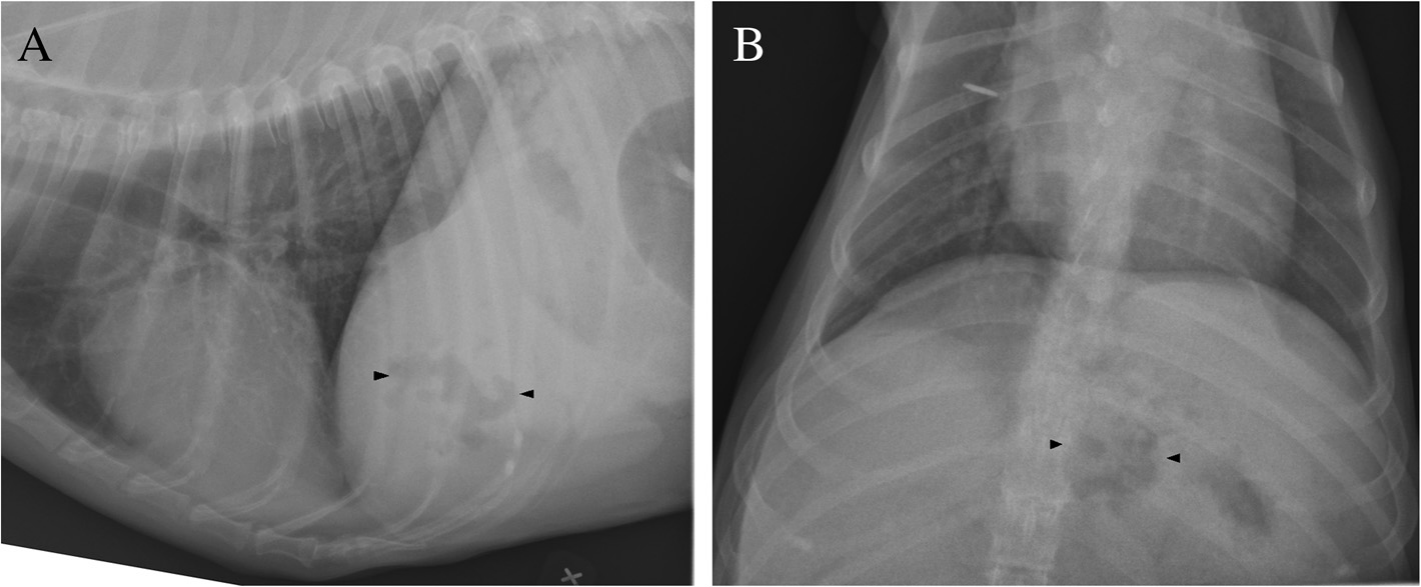
**Two – days post-biopsy radiographs of thorax and cranial abdomen.** Right lateral recumbent **(A)** and ventrodorsal projection **(B)** radiographs showing radiolucent gas (black arrowheads) in the mid ventral region of the liver.

Ultrasonography (linear probe VFX 13-5 MHz, Sonoline Antares, Siemens Medical Solution, Mountain View, USA) was done unsedated with the dog in lateral recumbency. A diffusely outlined focal area of severe hyperechogenicity associated with multiple reverberation artefacts was seen in the region of the previous liver biopsy and was considered to be intrahepatic gas (Figure 2). No evidence of pneumoperitoneum, pleural effusion or rupture of the biliary system or gastrointestinal tract was detected. Radiographs confirmed the focal region of radiolucent gas in the liver (Figure 3). Hepatic emphysema due to gas producing bacteria was strongly suspected. The clinical condition of the patient rapidly worsened and the dog died within 3 hours of readmission.

Post-mortem examination revealed acute hepatitis with hemorrhage, chronic hepatitis with dissecting fibrosis, and hepatic nodular hyperplasia. An extensive acute necrotizing hemorrhagic myositis was seen in the hind limb musculature. The intestinal mucosa was autolytic but a mild to moderate infiltration of lymphocytes and plasma cells was noted. Focal chronic pancreatitis and nodular hyperplasia, chronic glomerulonephritis with focal areas of emphysema in the renal cortex, and a chronic hemorrhagic cystitis was diagnosed, as was myxomatous valvular degeneration of the mitral valves and cardiac hemorrhage. Swabs were taken from the hind limb musculature for bacteriology and *Clostridium perfringens* was isolated in pure culture. Samples for bacteriology were not taken from the liver.

## Discussion

The culture of *C. perfringens* from the hindlimb muscles combined with areas of acute hepatic hemorrhages and gas production in the liver and kidneys strongly suggested bacteriemia and focal clostridial infection of the liver biopsy site in this dog. *C. perfringens* is a toxin producing commensal opportunistic anaerobic bacteria reported to be found in the bowel and heptobiliary tree of some individuals [8,9]. The bacteria can spread hematogenously, biliary or by wound penetration. It is possible in this case that the liver biopsy caused propagation of *C. perfringens* bacteria from the bile ducts due to communication between infected focus and blood vessels, resulting in systemic bacteriemia. More than 50% of biliary tract infections is due to *C. perfringens* in humans [10] and it has been shown to be one of the most common isolates in dogs and cats with hepatobiliary disease [11]. The diarrhea of the dog is a potential reason for the spread of *C. perfringens* to the liver by reflux from the intestine into the bile ducts. Bile culture was not carried out to confirm or exclude the presence of bacteria at the time of the biopsy procedure or post-mortem examination. Introduction of bacteria into the liver parenchyma by the biopsy needle is another possibility, although surgical preparation of the skin and the use of sterile biopsy needle makes this unlikely. Alternatively a hematogenous route of systemic spread of bacteria from the intestines is possible, with focal hepatic emphysema developing as a consequence of less resistance of the liver parenchyma following the mechanical damage by the biopsy needle in combination with chronic liver disease. Several risk factors for anaerobic blood stream infection preexisted in this dog including intestinal disease, chronic liver disease and heart disease [10,12]. Previous antimicrobial treatment at the referral clinic could potentially have disrupted the microenvironment of the gastrointestinal tract and enabled proliferation of *C. perfringens*. A penetrating wound of the hindlimb coinciding with the liver biopsy procedure and the gastrointestinal symptoms is another possible explanation for the bacterial infection that cannot be completely excluded, although no wound was found at post-mortem examination.

## Conclusion

This report shows a case of focal hepatic emphysema associated with percutaneous ultrasound-guided liver biopsy, suspected to be a result of a clostridial infection. Careful selection and preparation of patients, including identification of potential infection of the biliary or gastrointestinal tract, prior to liver biopsy is recommended.

## Competing interest

The authors declare that they have no competing interests.

## Authors’ contribution

FW did the hepatic biopsy, follow-up diagnostic imaging and is the main author. TH did the initial hepatic ultrasound and contributed with this part in the manuscript. MU reviewed the manuscript and contributed with ideas to the discussion. PE did the post-mortem examination and contributed with this part of the manuscript. CL conceived the idea of the manuscript and contributed substantially in all parts of the manuscript and was the main supervisor. All authors have read and approved the final manuscript.
